# Supplementary material for: Identification of Freezing-Responsive microRNAs and Their Targets in Chinese Jujube by Small RNA and Degradome Sequencing
Source: Int J Mol Sci. 2026 Sep 4;27(17):7912. doi: 10.3390/ijms27177912 (PMC13566890; doi:10.3390/ijms27177912)
Supplement: Supplementary file 1 [file ijms-27-07912-s001.zip › Supplementary Material S2/novel_miR_172.pdf]

| | | |
|---|---|---|
| Provisional ID | : | novel_miR_172 |
| Score total | : | 0.4 |
| Score for star read(s) | : | -1.3 |
| Score for read counts | : | 0 |
| Score for mfe | : | 0.1 |
| Score for randfold | : | 1.6 |
| Score for cons. seed | : | |
| Total read count | : | 112 |
| Mature read count | : | 103 |
| Loop read count | : | 9 |
| Star read count | : | 0 |

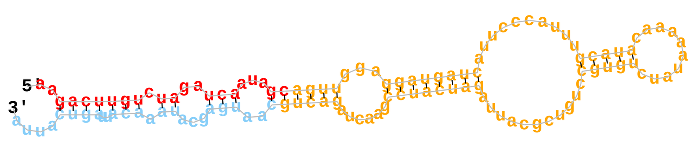

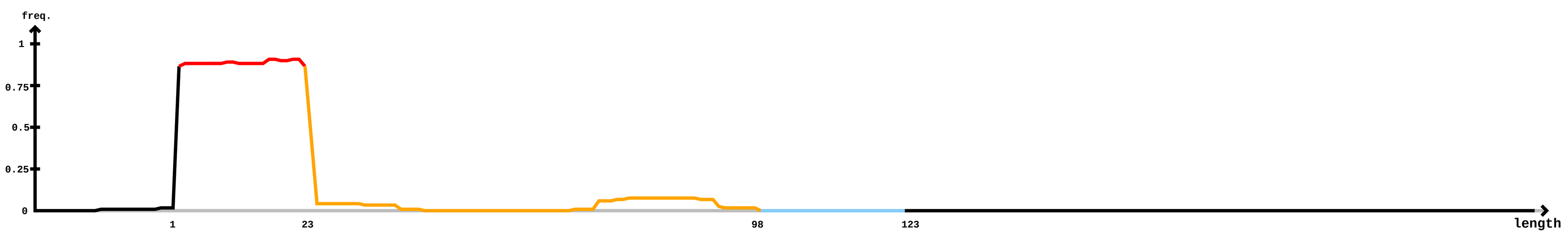


**Mature** **Star**

```
5'- uuaaauaacaaaucccucagcaaagacuugucuagaucaauagcaguuggaggaugaucauucccauuugcauacaaaaauaucugugccugucgcauuagaucauccgaacuagacugcaaugagcauaaacauuagucauuaucaacuucacuccauauucuuuuaaguugguucaauaagaagaauccgcuuccuaaguuagauucaaccacgauaggaaugugacacucauguuguuuauggggauu -3'
```

| read | reads | mm | sample |
|---|---|---|---|
| aaaucccucagcaaagacuuguc | 1 | 0 | LT-3 |
| gcaaagacuugucuagauca | 1 | 0 | LT-3 |
| aagacuugucuagaucaauag | 5 | 0 | LT-2 |
| aagacuugucuagaucaauagc | 41 | 0 | LCK-1 |
| aagacuugucuagaNcaauagc | 1 | 1 | LCK-1 |
| aagacuugucuagaucaauagc | 2 | 0 | LT-1 |
| aagacuugucuagaucaauagc | 52 | 0 | LCK-2 |
| agacuugucuagaucaauagca | 2 | 0 | LT-3 |
| ucuagaucaauagcaguuggagg | 1 | 0 | LT-2 |
| caauagcaguuggaggaugauc | 1 | 0 | LT-3 |
| caauagcaguuggaggaugauc | 2 | 0 | LT-1 |
| agcaguuggaggaugaucauuc | 1 | 0 | LT-1 |
| ccugucgcauuagaucauccg | 1 | 0 | LT-3 |
| ucgcauuagaucauccgaac | 5 | 0 | LCK-3 |
| ucgcauuagaucauccgaacuagacug | 1 | 0 | LT-1 |
| cCuuagaucauccgaacu | 1 | 1 | LT-2 |
| uuagaucauccgaacuagacug | 1 | 0 | JCK-3 |
